# Hypermethylation of *Smad7* in CD4^+^ T cells is associated with the disease activity of rheumatoid arthritis

**DOI:** 10.3389/fimmu.2023.1104881

**Published:** 2023-02-09

**Authors:** Yiping Hu, Bihua Xu, Juan He, Hongying Shan, Gengmin Zhou, Deli Wang, Lu Bai, Hongxi Shang, Liping Nie, Fan Pan, Hui Yao Lan, Qingwen Wang

**Affiliations:** ^1^ Department of Rheumatism and Immunology, Peking University Shenzhen Hospital, Shenzhen, Guangdong, China; ^2^ Shenzhen Key Laboratory of Immunity and Inflammatory Diseases, Shenzhen, Guangdong, China; ^3^ Department of Bone and Joint Surgery, Peking University Shenzhen Hospital, Shenzhen, Guangdong, China; ^4^ Department of Sports Medicine, Peking University Shenzhen Hospital, Shenzhen, Guangdong, China; ^5^ Department of Bone and Joint Surgery, Shenzhen Second People’s Hospital, The First Affiliated Hospital of Shenzhen University, Shenzhen, Guangdong, China; ^6^ Department of Clinical Laboratory, Peking University Shenzhen Hospital, Shenzhen, Guangdong, China; ^7^ Center for Cancer Research, Shenzhen Institutes of Advanced Technology, Chinese Academy of Sciences, Shenzhen, Guangdong, China; ^8^ Department of Medicine and Therapeutics, Li Ka Shing Institute of Health Sciences, and Lui Che Woo Institute of Innovative Medicine, The Chinese University of Hong Kong, Hong Kong, Hong Kong SAR, China; ^9^ Guangdong-Hong Kong Joint Laboratory for Immunological and Genetic Kidney Disease, Department of Pathology, Guangdong Academy of Medical Science, Guangdong Provincial People’s Hospital, Guangzhou, China

**Keywords:** hypermethylation, Smad7, rheumatoid arthritis, DNA methyltransferase, methyl-CpG binding domain

## Abstract

**Background:**

Smad7 is protective in a mouse model of rheumatoid arthritis. Here we investigated whether Smad7-expressing CD4^+^ T cells and the methylation of *Smad7* gene in CD4^+^ T cells contribute to the disease activity of RA in patients.

**Methods:**

Peripheral CD4^+^ T cells were collected from 35 healthy controls and 57 RA patients. Smad7 expression by CD4^+^ T cells were determined and correlated with the clinical parameters of RA including RA score and serum levels of IL-6, CRP, ESR, DAS28-CRP, DAS28-ESR, Swollen joints and Tender joints. Bisulfite sequencing (BSP-seq) was used to determine the DNA methylation in Smad7 promoter (-1000 to +2000) region in CD4^+^ T cells. In addition, a DNA methylation inhibitor, 5-Azacytidine (5-AzaC), was added to CD4^+^ T cells to examine the possible role of Smad7 methylation in CD4^+^ T cell differentiation and functional activity.

**Results:**

Compared to the heath controls, Smad7 expression was significantly decreased in CD4^+^ T cells from RA patients and inversely correlated with the RA activity score and serum levels of IL-6 and CRP. Importantly, loss of Smad7 in CD4^+^ T cell was associated with the alteration of Th17/Treg balance by increasing Th17 over the Treg population. BSP-seq detected that DNA hypermethylation occurred in the Smad7 promoter region of CD4^+^ T cells obtained from RA patients. Mechanistically, we found that the DNA hypermethylation in the Smad7 promoter of CD4^+^ T cells was associated with decreased Smad7 expression in RA patients. This was associated with overreactive DNA methyltransferase (DMNT1) and downregulation of the methyl-CpG binding domain proteins (MBD4). Inhibition of DNA methylation by treating CD4^+^ T cells from RA patients with 5-AzaC significantly increased Smad7 mRNA expression along with the increased MBD4 but reduced DNMT1 expression, which was associated with the rebalance in the Th17/Treg response.

**Conclusion:**

DNA hypermethylation at the Smad7 promoter regions may cause a loss of Smad7 in CD4^+^ T cells of RA patients, which may contribute to the RA activity by disrupting the Th17/Treg balance.

## Introduction

Rheumatoid arthritis (RA) is characterized as a chronic autoimmune disease that affects the joints. The primary clinical feature of RA is a long-term chronic inflammation of the joints, leading to the bone erosion, cartilage destruction, and disability (1, 2). Several cells, including dendritic cells (DCs), T cells, B cells, neutrophils, macrophages, fibroblasts, and osteoclasts can interact each other to initiate and maintain this inflammatory process (1, 3). CD4^+^ T cells account for a large proportion of T cells and play vital role in mediating inflammation of the synovial tissue in RA patients. During immune response, naïve CD4^+^ T cells could be activated and distinguished into diverse T helper (Th) cell subsets (4). However, the specific role and regulatory mechanisms of CD4^+^ T cells in RA patients remain unclear (4, 5).

Mothers against decapentaplegic homolog 7 (Smad7) is an important inhibitory factor of the transforming growth factor-β (TGF-β) signaling pathway, which exerts an essential role in the prevention and treatment of various diseases (6–8). The absence of Smad7 results in activation of TGF-β/Smad3-IL-6 and NF-κB pathways and leads to synovial inflammation in RA patients and collagen-induced arthritis (CIA), which is associated with the imbalance in TH17/Treg response by a 2.8-fold increase in the Th17/Treg ratio (9). On the other hand, intraarticular overexpression of Smad7 ameliorated CIA (10). These studies suggest a protective role of Smad7 in pathogenesis of RA. However, the underlying mechanisms through which loss of Smad7 under RA conditions remains unclear, which was investigated in the present study.

DNA methylation is a prime epigenetic modification form responsible for regulating numerous cellular processes, including chromosome stability, transcription and embryonic development (11, 12). DNA methylation occurs mainly, but not exclusively, on cytosine residues that lie in Cytosine-phosphate-Guanine (CpG) dinucleotides in eukaryotes (12). DNA methyltransferase (DNMT) family members are the main regulators of the genomic CpG islands methylation (13). Furthermore, methyl-CpG-binding domain (MBD) family members can influence the activity of genes by binding primarily to the methylated CpG region *via* MBD site (14). It has been scrutinized that DNA methylation-dependent epigenetic modification of *Smad7* gene promoter decreases its expression in rat hepatic myofibroblasts (15). However, the Smad7 gene methylation level in CD4^+^ T cells of RA patients and the potential regulating mechanisms are still unclear.

In this study, we first examined Smad7 expression in peripheral CD4^+^ T cells obtained from RA patients and corrected CD4^+^ T cell Smad7 with the RA activity and immune responses. Then, we investigated the mechanism through which Smad7 is downregulated by focusing on DNA methylation of *Smad7* promoter in CD4^+^ T cells.

## Materials and methods

### Patients

Peripheral blood samples were obtained from a total of 92 individuals, including 35 healthy controls (HC) and 57 RA patients. All patients fulfilled the American College of Rheumatology(ACR 1987) criteria for RA (16) and the clinical characteristics were shown in **Supplementary STable 1**. Eleven active RA patients were followed longitudinally. All patients had received conventional disease modifying anti-rheumatic drug (cDMARDs) and achieved significant improvement in the disease activity. Blood samples were obtained before and 3 months after the treatment. The Disease Activity Score in 28 joints (DAS28) was used to evaluated the disease activity of RA (remission: DAS28<2.6, low disease activity: 2.6≤DAS28<3.2, intermediate disease activity: 3.2≤DAS28≤ 5.1, and high disease activity: DAS28>5.1) (17). A written consent was attained from all subjects. The study was approved by Peking University Shenzhen Hospital and performed following their accredited ethical guidelines (2020–007).

Synovial biopsy tissues were obtained from 13 RA patients and 5 controls from the subjects without rheumatic disease and from the accidental injury following knee surgery (3 females and 2 males with mean age of 41.8 ± 13.5 years) for immunofluorescent staining as described below.

### Immunofluorescence

The freshly isolated Peripheral blood mononuclear cells (PBMCs) were collected from heparinized peripheral blood through density gradient centrifugation using the Ficoll-PaqueTM PREMIUM sterile solution (GE Healthcare Bio-Sciences AB, Sweden). Positive selection method using magnetic beads was utilized for CD4^+^ T cells isolation as per the manufacturer’s instructions (MACS Miltenyi Biotec, Germany). The purity of isolated cells was confirmed by flow cytometry with more than 85% CD4^+^ population. The CD4^+^ T cells were then fixed with the 95% ethanol and stained with anti-Smad7 antibody (eBioscience, United States) by immunofluorescence.

Frozen synovial biopsy tissues obtained from RA patients were stained for two-color immunofluorescence using the PE-labeled anti-human CD4 antibody and FITC labeled anti-human Smad7 antibody (eBioscience, United States), followed by DAPI for nuclear staining. Finally, a total of 10 consecutive×40 high-power fields/sections were counted under fluorescence microscope (Axioplan2 imaging, Carl Zeiss, Germany) equipped with an eyepiece graticule (0.0625mm2) to estimate the positive cells (single, double, or triple). Data expressed as cells per mm^2^.

### Bisulfite sequencing

Bisulfite sequencing PCR (BSP), next-generation sequencing technique, was employed to identify gene-specific DNA methylation. Brief summary of the method is as follows:BSP-specific primers were adapted through the online MethPrimer software and listed in **Stable 2** (a total of three pairs of primers were designed for sequencing analysis). Zymo EZ DNA Methylation-Gold Kit (Zymo Research, Irvine, CA, USA) was utilized for the conversion of genomic DNA (1μg). KAPA HiFi HotStart Uracil+ ReadyMix PCR Kit (Kapa Biosystems, Wilmington, MA, USA) was used for PCR amplification (35 cycles) from elution products (one-twentieth) as templates. BSP products of multiple genes for each sample were pooled uniformly, and 5’-phosphorylated and 3’-dA-tailed fragments were ligated to a barcoded sequencing adapter by utilizing the T4 DNA ligase (NEB). Illumina platform was used for sequencing barcoded libraries from all samples (the sequencing service is completed by Shenzhen Ace Gene Technology Co., Ltd.).

### RNA isolation and qRT-PCR analysis

Total RNA was extracted from isolated CD4^+^ T cells using RNA purification kits purchased from Omega (Omega Bio-Tek, Inc. R6831-01, USA). qRT-PCR assays were done using a Light Cycler 96 system (Bio-Rad, USA). PCR setup was as follows: pre-denaturation at 50°C for 2 min and 95°C for 10 min, with subsequent 40 cycles at 95°C, 60°C and 72°C for 15s, 20s and 30s, respectively. The primer sequences used for qRT-PCR are shown in **STable 3**.

### Treatment of CD4^+^ T cells with 5-AzaC

The isolated CD4^+^ T cells were stimulated with phytohemagglutinin (PHA) (Shanghai Maokang Biotechnology Co., Ltd. Shanghai, China) and dealt with 2μM 5-AzaC (Sigma-Aldrich, USA) and 50 units/mL rIL-2 (R&D Systems, USA) in RPMI 1640/10% FBS for an additional 72h. After stimulation, the cells were rinsed and used for flow cytometric analysis.

### Flow cytometry analysis

PBMCs were isolated from RA patients using density-gradient centrifugation on Ficoll-Paque. The cell pellets were washed and resuspended with PBS containing 1% FBS. Then, the cells were incubated with FITC-conjugated CD4, PE-Cy5-conjugated CD3, APC-conjugated CD25 and PE-conjugated CD127(all from BD Pharmingen, USA). For intracellular cytokine staining, the cells were pretreated with Cell Activation Cocktail (Thermo) for 6h. Then, cells were washed and stained with PE-conjugated CD4 and AF750-conjugated CD3 antibodies (all from BD Pharmingen, USA). After fixed and permeabilized with Transcription Factor Buffer Set (BD Pharmingen, USA), the cells were stained with AF700-conjugated IL-17A (Biolegend, USA), FITC-conjugated IFN-γ and PE-Cy7 conjugated IL-4 antibodies (all from BD Pharmingen, USA). For Foxp3 staining, cells were fixed and permeabilized, and then stained with BV421 conjugated Foxp3 antibody (Biolegend, USA). Multiparameter flow cytometry (DxFLEX, Beckmancoulter) and FlowJo software (Tree Star) were adopted for the data analysis of stained cells.

Cytometric bead array (CBA) was used to measure IL-6, IFN-γ, IL-4, IL-17A and IL-10 levels in serum from RA patients or health controls. The measurement was performed using Aimplex Human Th1/Th2/Th17 14-plex cytokine kit (Quanto Bio, China) according to the manufacture’s instruction.

### ELISAs

Enzyme-linked immunosorbent assay (Elisa) was used for detecting cytokine concentrations from the cultured CD4^+^ T cells supernatants. The isolated CD4^+^ T cells were stimulated with phytohemagglutinin (PHA) (Shanghai Maokang Biotechnology Co., Ltd. Shanghai, China) and dealt with 2μM 5-azaC (Sigma-Aldrich, USA) and 50 units/mL rIL-2 (R&D Systems, USA) in RPMI 1640/10% FBS for an additional 72h. Then, the supernatants were collected for ELISA (MILLIPLEX MAP Kit, HSTCMAG-28SK, Merck KGaA, UAS) following the ILISA kit instructions.

### Statistical analysis

Normally distributed data are presented as the mean ± SD. Non-normally distributed data were presented as median ± interquartile range. Student’s t-test was used for independent two-group analyses. Paired data for eleven patients before and after treatment were compared using a paired t-test. Spearman’s rank correlation test was used to determine correlations. A p-value < 0.05 was considered statistically significant.

## Results

### Loss of Smad7 in CD4^+^ T cells is associated with high disease activity in RA patients

**Figure 1:**
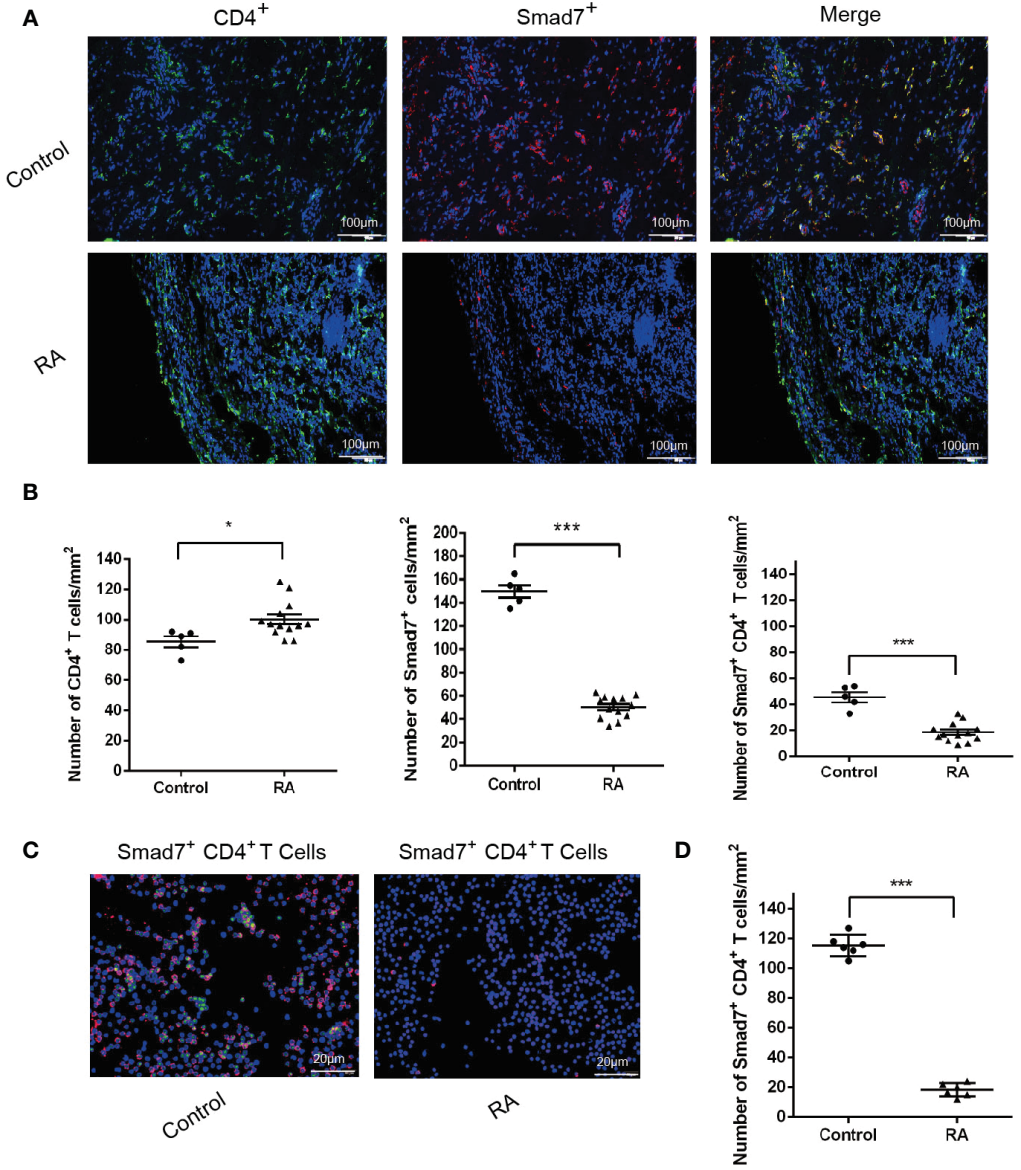
Immunofluorescence detects expression of Smad7 by CD4^+^ T cells from synovial tissues and peripheral blood of RA patients: **(A, B)** Two-color immunofluorescence and quantitative analysis of CD4^+^, Smad7^+^ and Smad7^+^ CD4^+^ cells in the synovial tissues of RA patients and health controls. **(C, D)** CD4^+^ T cells were sorted from peripheral of RA patients and health controls. More than 85% cells were confirmed to be CD4^+^ T cells by flow cytometry. The Smad7 immunofluorescence and quantitative analysis were conducted in the sorted CD4^+^ T cells from RA patients and health controls. Each dot represents one individual and data are the mean ± SD. *P < 0.05, ***P < 0.001 compared between groups.

**Figure 2:**
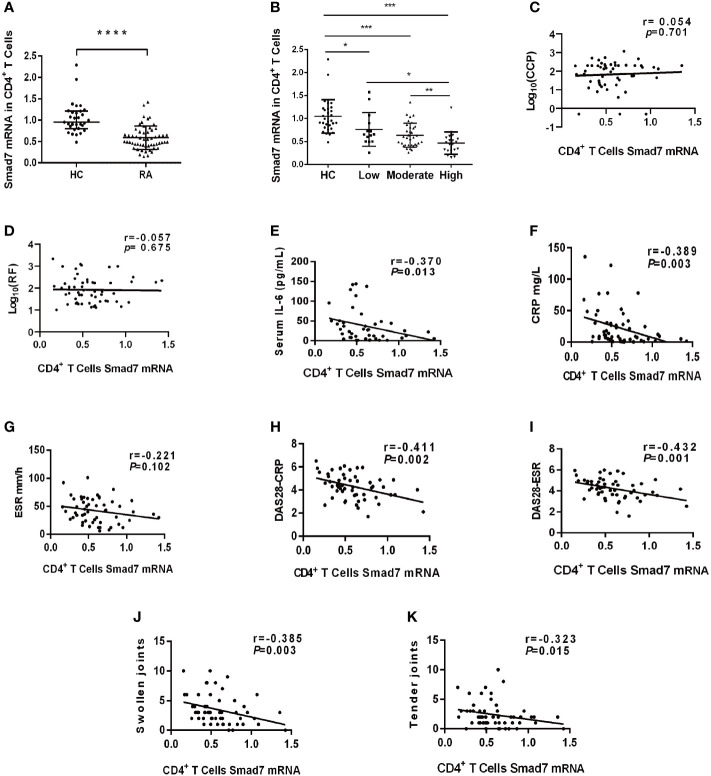
The expression of Smad7 in peripheral CD4^+^ T cells and its correlation with clinical indexes in RA patients. **(A)** Expression of Smad7 mRNA in peripheral CD4^+^ T cells of RA patients (n=57) and health controls (n=35). **(B)** Levels of Smad7 mRNA in CD4^+^ T cells of RA patients with different disease activities. **(C, D)** Correlation of CD4^+^ T cell Smad7 mRNA level with serum levels of CCP or RF in RA patients (n=56). **(E–K)** Correlation of CD4^+^ T cell Smad7 mRNA level with serum levels of IL-6, CRP, ESR, DAS28-CRP, DAS28-ESR, Swollen joints and Tender joints in RA patients (n=56). Each dot represents one individual and data are mean ± SD. *P < 0.05, **P < 0.01, ***P < 0.001, ****P < 0.0001 compared between groups.

**Figure 3:**
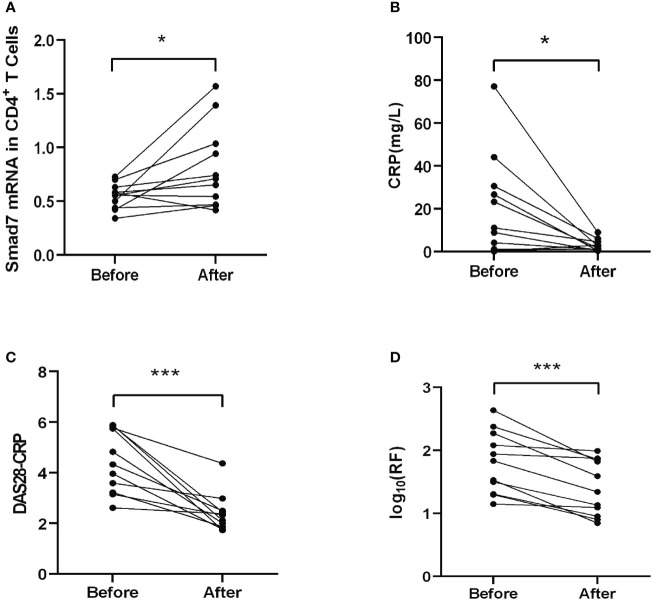
Therapeutic effect on Smad7 mRNA expression of the peripheral CD4^+^ T cells and the clinical indexes in RA patients. **(A)** The mRNA level of Smad7 in peripheral CD4^+^ T cells of RA patients before and after treatment. **(B)** The serum levels of CRP before and after treatment. **(C)** The DAS28-CRP score before and after treatment. **(D)** The serum levels of RF before and after treatment. *P < 0.05, ***P < 0.001. n=11.

Two-color immunofluorescence was used to detect the Smad7 expression by CD4^+^ T cells of synovial tissues from both RA patients and the health controls. We found that CD4^+^ T cells infiltrating the synovial tissues of RA patients were significantly increased, however, expression of Smad7 by CD4^+^ T cells (Smad7^+^ CD4^+^) were largely reduced in RA patients (**Figures 1A, B**). Similar results were also observed in peripheral blood CD4^+^ T cells in which high levels of Smad7-expressing CD4^+^ T cells seen in the health controls were almost lost in RA patients (**Figures 1C, D**). qRT-PCR also confirmed this notion that Smad7 mRNA expression by the peripheral CD4^+^ T cells was much lower in RA patients compared to the health controls (**Figure 2A**). Furthermore, we divided the RA patients into three groups based on their disease activities and found that lower levels of Smad7 mRNA expression in the peripheral CD4^+^ T cells were associated with higher disease activities and inversely correlated with serum levels of IL-6, CRP, DAS28-CRP, DAS28-ESR, Swollen joints and Tender joints in RA patients (**Figures 2B, E, F, H–K**). However, there was no significant correlation between the Smad7 mRNA level and serum levels of RF, ESR, and anti-CCP antibody (**Figures 2C, D, G**). Interestingly, in those RA patients that were responsive to the treatment, expression of Smad7 by the peripheral CD4^+^ T cells was significantly increased (**Figure 3A**), which was also accompanied by decreased serum levels of CRP, RF, and DAS28-CRP score (**Figures 3B–D**). These results indicate that Smad7-expressing CD4^+^ T cells may predict the disease activity in RA patients.

### Loss of Smad7 in CD4^+^ T cells is associated with Th17/Treg imbalance and aberrant cytokine expression in RA patients

**Figure 4:**
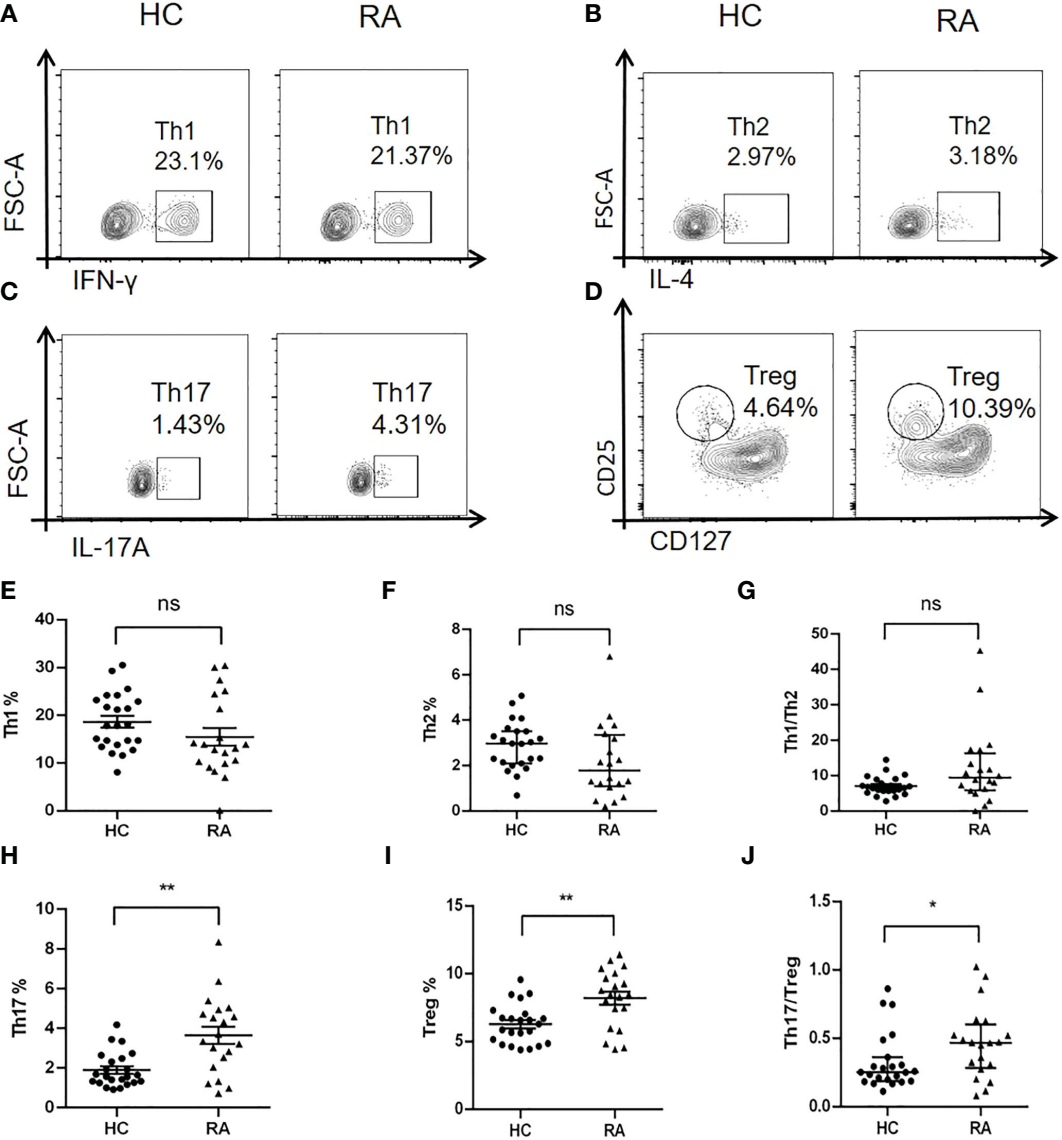
Multiparameter flow cytometry analysis of peripheral CD4^+^ T cell subtypes in RA patients and health controls. **(A, E)** Representative flow-cytometric histogram and quantitative analysis of the Th1 subset (CD4^+^IFN-γ^+^). **(B, F)** Representative flow-cytometric histogram and quantitative analysis of the Th2 subset (CD4^+^IL-4^+^). **(C, H)** Representative flow-cytometric histogram and quantitative analysis of the Th17 subset (CD4^+^IL-17A^+^). **(D, I)** Representative flow-cytometric histogram and quantitative analysis of the Treg subset (CD4^+^CD25^+^CD127^-^). **(G)** The ratio of Th1/Th2. **(J)** The ratio of Th17/Treg. Each dot represents one individual and data are the mean ± SD. *P < 0.05, **P < 0.01. ns, not significant.

**Figure 5:**
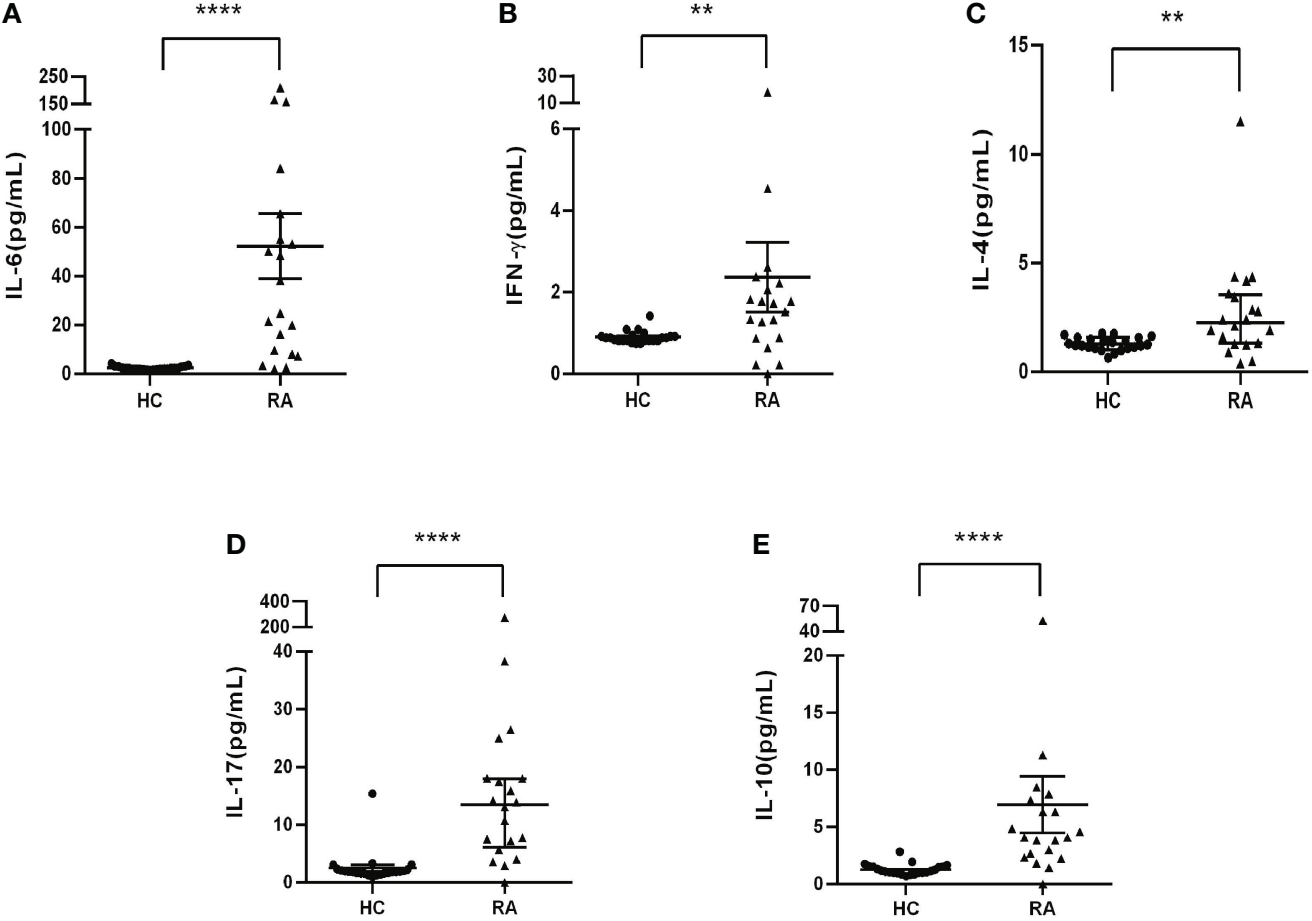
Serum levels of cytokines in RA patients and health controls by CBA. **(A)** Levels of IL-6. **(B)** Levels of INF-γ. **(C)** Levels of IL-4. **(D)** Levels of IL-17. **(E)** Levels of IL-10. Each dot represents one individual and data are mean ± SD. **P < 0.001, ****P < 0.0001.

TGF-β/Smad signaling plays a critical role in regulating differentiation of naïve CD4^+^ T cells into distinct effector lineages (18). As an important inhibitory regulator of TGF-β signaling, Smad7 may also involve in regulating CD4^+^ T cells differentiation. We first analyzed the phenotypic changes of CD4^+^ T cells in peripheral blood of RA patients and health controls and found that there was no significant alteration in the Th1 and Th2 populations as well as the Th1/Th2 ratio in RA patients when compared with HC group (**Figures 4A, B, E–G**). However, a significant increase in Th17 population was found in RA patients, which was associated with the imbalance of Th17/Treg by shifting Th17 over Treg population, although the Treg cells were also increased in RA patients (**Figures 4C, D, H–J**). The imbalance in the Th17/Treg responses also contributed to aberrant cytokine expression as demonstrated by a significant increase in serum levels of IL-6, IFN-γ, IL-4 and IL-17 and IL-10 in RA patients (**Figure 5**).

### Loss of Smad7 is associated with hypermethylation of *Smad7* promoter in peripheral CD4^+^ T cells in RA patients

**Figure 6:**
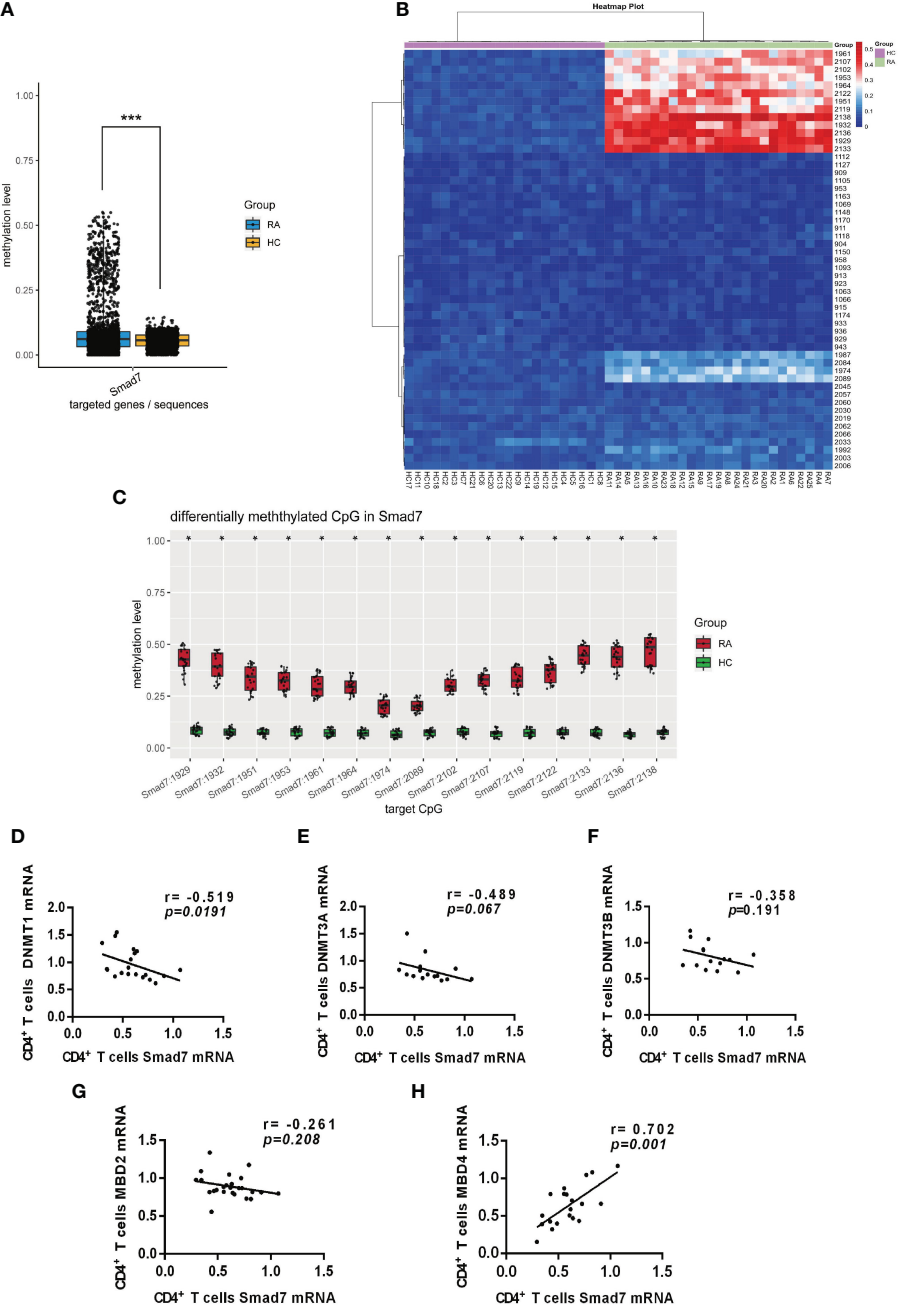
*Smad7* promoter methylation of the peripheral CD4^+^ T cells from RA and health controls. **(A)** The mean methylation at the *Smad7* promoter (-1000 to +2000bp) in CD4^+^ T cells from RA (n=25) and health controls (n=22) groups (*P=*7.1411e-05). **(B)** Heatmap shows that hypermethylation at the CG pairs (For the convenience of expression and counting, when we draw the heatmap, we count the positions according to the direction of the positive strand of the genome. That is, the position 1k downstream of the transcription start position of the gene is recorded as 1, and the position 2k upstream of the transcription start position is recorded as 3000, and counted according to this rule and marked in the picture. +927 (1929), +930 (1932), +949 (1951), +951 (1953), +959 (1961), +962 (1964), +972 (1974), +1087 (2089), +1100 (2102), +1105 (2107), +1117 (2119), +1120 (2122), +1131 (2133), +1134 (2136), +1136 (2138)) in the RA patients compared to health controls. **(C)** The average methylation status of the 15 CG pairs (positions: +927 (1929), +930 (1932), +949 (1951), +951 (1953), +959 (1961), +962 (1964), +972 (1974), +1087 (2089), +1100 (2102), +1105 (2107), +1117 (2119), +1120 (2122), +1131 (2133), +1134 (2136), +1136 (2138)) is significantly increased in CD4^+^ T cells from RA patients compared to the health individuals. **(D–F)** Correlation of Smad7 mRNA with DNMT1, DNMT3A, or DNMT3B mRNA in CD4^+^ T cells in RA patients. CD4^+^ T cells from RA patients. **(G, H)** Correlation of Smad7 mRNA with MBD2 or MBD4 mRNA in CD4^+^ T cells from RA patients. Each dot represents one individual. *P < 0.05.<0.05, ***P < 0.05.<0.0001.

DNA methylation is an epigenetic mechanism that regulates gene expression (19). We hypothesized that loss of Smad7 in CD4^+^ T cells may be associated with hypermethylation on the *Smad7* promoter and thus analyzed the methylation status in 3000bp of the *Smad7* promoter (-1000 to +2000bp) using BSP sequencing. We found that DNA methylation on the Smad7 promoter occurred at +927 to +1136 CG pairs site. There was a significant variation in the Smad7 promoter methylation status in the peripheral CD4^+^ T cells between RA patients and health controls (**Figure 6A**). The heatmap showed that the amount of methylation at the CG pairs [positions: +927 (1929), +930 (1932), +949 (1951), +951 (1953), +959 (1961), +962 (1964), +972 (1974), +1087 (2089), +1100 (2102), +1105 (2107), +1117 (2119), +1120 (2122), +1131 (2133), +1134 (2136), +1136 (2138)] was considerably increased in the RA patients compared to the health controls (**Figure 6B**). The average methylation status of the above 15 CG pairs was notably exceeded in the peripheral CD4^+^ T cells in RA patients than that found in the health individuals (**Figure 6C**).

It is well known that DNA methylation is regulated by DNA methyltransferases (DNMTs) (20, 21). We then examined expression of DNMTs on CD4^+^ T cells and found that among DNMT1, DNMT3A, and DNMT3B, only DNMT1 mRNA expression was negatively correlated with the expression of Smad7 in CD4^+^ T cells (**Figures 6D–F**). It is also reported that the methyl-CpG binding domain (MBD) family members can bind to methylated DNA regions and regulate the target gene transcription (22). We then examined expression of MBD family members and found that the MBD4 but not MBD2 mRNA positively correlated with the expression of Smad7 (**Figures 6G, H**). Thus, DNA hypermethylation in the *Smad7* promoter in CD4^+^ T cells may be associated with decreased expression of Smad7 in RA patients, which may be regulated by DMNT1 and MBD4.

### Inhibition of Smad7 methylation restores the balance of Th17/Treg response *in vitro*

**Figure 7:**
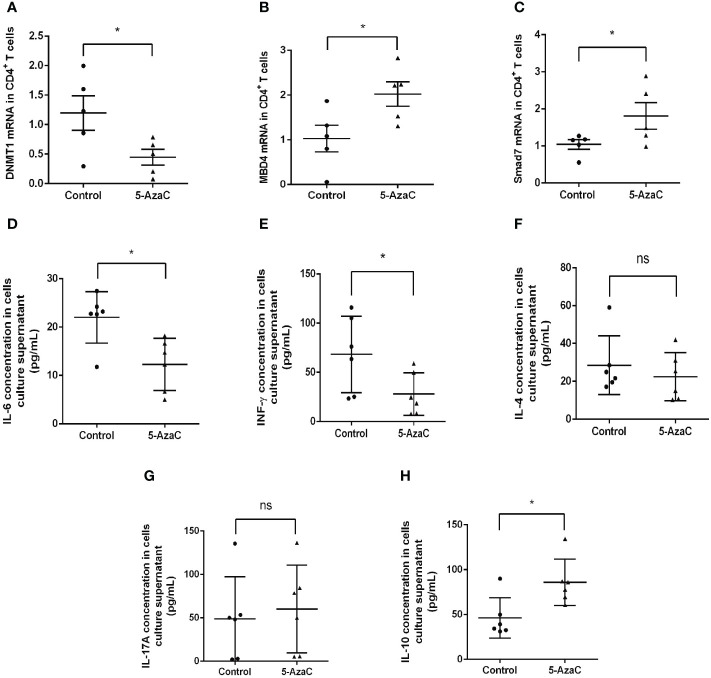
*In-vitro* effects of 5-AzaC on expression of DNMT1, MBD4, Smad7 mRNA and T cell cytokines in CD4^+^ T cells from RA patients by real-time PCR. The methylation effects were assessed in CD4^+^ T cells after stimulation with PHA and IL-2 in the presence or of 5-AzaC (2μM). **(A)** The DNMT1 mRNA. **(B)** The MBD4 mRNA. **(C)** The Smad7 mRNA. **(D–H)** ELISA for IL-6, INF-γ, IL-4, IL-17A, and IL-10. Each dot represents one individual and data are mean ± SD. *P < 0.05. ns, not significant.

We next investigated the potential role of Smad7 methylation in T cell responses by treating the peripheral blood CD4^+^ T cells from RA patients with 5-AzaC, a DNA methylation inhibitor. Firstly, we examined the mRNA levels of DNMT1, MBD4 and Smad7 respectively. Results showed the DNMT1 mRNA level was significantly decreased in CD4^+^ T cells treated with 5-AzaC (**Figure 7A**). In contrast, that treatment with 5-AzaC significantly increased the MBD4 and Smad7 mRNA levels in CD4^+^ T cells compared with control group (**Figures 7B, C**).

**Figure 8:**
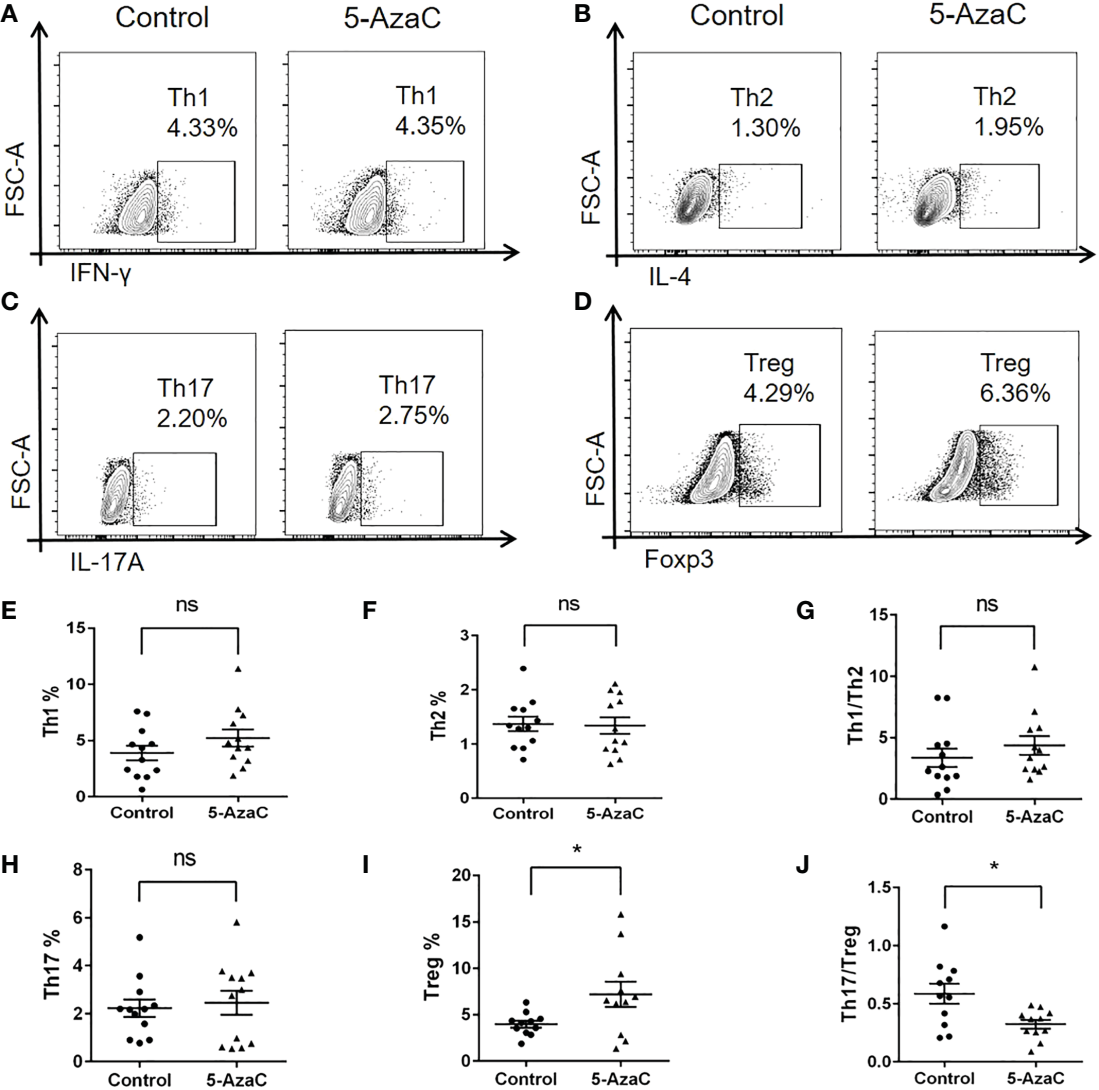
*In-vitro* effects of 5-AzaC on subsets of CD4^+^ T cells from RA patients by multiparameter flow cytometry. The differentiation of CD4^+^ T cells isolated from peripheral blood of RA patients was assessed after stimulation with PHA and IL-2 in the presence or absence of 5-AzaC (2μM). **(A, E)** Representative flow-cytometric histogram and quantitative analysis of the Th1 subset (CD4^+^IFN-γ^+^). **(B, F)** Representative flow-cytometric histogram and quantitative analysis of the Th2 subset (CD4^+^IL-4^+^). **(C, H)** Representative flow-cytometric histogram and quantitative analysis of the Th17 subset (CD4^+^IL-17A^+^). **(D, I)** Representative flow-cytometric histogram and quantitative analysis of the Treg subset (CD4^+^Foxp3^+^). **(G)** The Th1/Th2 ratio. **(J)** The Th17/Treg ratio. Each dot represents one individual and data are the mean ± SD for groups of 12 samples *P < 0.05. ns, not significant.

Importantly, we also found that inhibition of *Smad7* methylation significantly upregulated Treg without altering the Th1, Th2 and Th17 immune responses (**Figures 7D–H**, **8**). Indeed, compared to the CD4^+^ T cells from RA patients where Th17 overrided the Treg response (**Figure 4J**), treatment with 5-AzaC resulted in the shift from Th17 to Treg responses (**Figures 8C, D, H–J**) and increased Treg cytokine IL-10 while suppressing IL-6 and INF-γ release by the CD4^+^ T cells from RA patients (**Figure 7D, E, H**). These results suggested that the DNA methylation may be a mechanism associated with the loss of Smad7 in CD4^+^ T cells in RA patients. Thus, aberrant expression of Smad7 may impair the balance in the Th17/Treg immune responses in RA patients.

## Discussion

We previously reported that expression of Smad7 is decreased in the synovial tissues of RA patients (9). We also reported a protective role for Smad 7 in RA, which is demonstrated in a mouse model of CIA in which mice lacking Smad7 gene develop much more severe joint injury with massive inflammation and Th17 over the Treg immune responses in the joint tissues (9). In contrast, intraarticular overexpression of Smad7 ameliorates experimental arthritis (10). Given that CD4^+^ T cells play an important role in the initiation and progression of RA (23), this study investigated Smad7 expression in CD4^+^ T cells in RA patients. We found that Smad7 was lost in peripheral CD4^+^ T cells from RA patients which inversely correlated with the RA disease activity score and serum levels of IL-6 and CRP. Importantly, we also uncovered that loss of Smad7 in CD4^+^ T cells was associated with dysregulation of Th17/Treg by enhancing Th17 over Treg response, providing the first clinical evidence for the potential role of Smad7 in CD4^+^ T cell-mediated RA. This clinical finding is consistent with known role of Smad7 in T cell response in a mouse model of CIA in which deletion of Smad7 promotes Th17 over Treg response (9). It is well known that the Th17 cells are the main effector cells in the pathogenesis of RA, whereas Treg cells are protective and the imbalance between Th17 and Treg cells is critical in the pathogenesis of RA (24–27). Consistent with this notion, we also found that the imbalance of Th17/Treg in the CD4^+^ T cells was associated with loss of Smad7. It is well established that TGF-β plays a critical role in CD4^+^ T cell differentiation and functions (28, 29). Signals of the TGF-β are transduced by intracellular receptor-associated Smads including Smad2 and Smad3. Smad7 is an inhibitory Smad and acts as a negative regulator of TGF-β signaling (30). TGF-β promotes the differentiation of Treg cells *via* a Foxp3-dependent mechanism (31, 32), whereas, TGF-β also induces Th17 differentiation in combination with IL-6 *via* the RORγt-dependent pathway in patients with RA (33). It is highly possible that loss of Smad7 in CD4^+^ T cells may impair the balance of TGF-β/Smad signaling with overreactive Smad3, which, together with IL-6, promotes Th17 response, resulting in the imbalance of Th17/Treg in RA patients. Thus, Smad7 specifically expressed by CD4^+^ T cells may play a role in the pathogenesis of RA by rebalancing the Th17/Treg immune response *via* by inhibiting TGF-β/Smad3 signaling, which requires further investigation.

A novel and significant finding from this study is the identification of DNA methylation as a mechanism responsible for the loss of Smad7 from CD4^+^ T cells in RA patients. This was supported by the finding that CD4^+^ T cells from RA patients exhibited DNA hypermethylation on the promoter region of Smad7 gene. Furthermore, inhibition of methylation with 5-AzaC was able to restore Smad7 expression in CD4^+^ T cells transcriptionally, and therefore suppressed Th17 while promoting Treg responses while increasing the IL-10 release. It is well accepted that epigenetic modifications play a crucial role in the pathogenesis of RA (34, 35). DNA methylation also plays a critical role in T cell development, differentiation, and functions and is also considered as a therapeutic target for RA (36). Hypermethylation on the gene promoters is generally accompanied by the transcriptional inhibition of downstream target genes (37, 38). It is well known that DNA methylation is mediated by DNMTs but is protected by MBD (39, 40), In the present study, we also found that the hypermethylation occurred in the Smad7 gene promoter (-1000 to +2000) in human peripheral RA CD4^+^ T cells, which was associated with increased DNMT1, but not DNMT3A and DNMT3B, while suppressing MBD4 but not MBD2 expression by CD4^+^ T cells. In contrast, blockade of methylation with 5-AzaC restored Smad7 expression in CD4^+^ T cells from RA patients, which was associated with inhibition of DNMT1 while increasing MBD4 expression by peripheral CD4^+^ T cells in RA patients. However, it should be pointed out that although DNMT1 was found to be associated with hypermethylation of Smad7, it cannot exclude the regulatory role of DNMT3A and DNMT3B on Smad7 expression. In addition, because 5-azaC is a broad-spectrum methyltransferase inhibitor, it is highly possible that the use of 5-azaC may also alter other genes that regulate Smad7 expression.

In summary, Smad7 was lost in CD4^+^ T cells in RA patients, which was associated with the development of RA activity and the imbalance of Th17/Treg. DNA hypermethylation at the Smad7 promoter region may be a mechanism responsible for the loss of Smad7 in CD4^+^ T cells in RA patients. Results from this study also suggested that Smad7 may play a role in rebalancing Th17/Treg responses in RA patients.

## Data availability statement

The sequencing data presented in this study can be found in online repositories. The names of the repository/repositories and accession number(s) can be found below: NCBI, PRJNA930061.

## Ethics statement

The study was approved by Peking University Shenzhen Hospital and performed following their accredited ethical guidelines (2020–007). The patients/participants provided their written informed consent to participate in this study. Written informed consent was obtained from the individual(s) for the publication of any potentially identifiable images or data included in this article.

## Author contributions

All authors were involved in drafting the article or revising it critically for important intellectual content, and all authors approved the final version to be published. YH and BX had full access to all of the data in the study and take responsibility for the integrity of the data and the accuracy of the data analysis. Study conception and design, QW, H-YL and FP. Acquisition of data, YH, HY-L, JH, LN and GZ. DW, LB and HXS had collected surgical specimens. All authors contributed to the article and approved the submitted version.

## Supplementary material

Supplementary Figure 1 Smad7 promoter methylation sequencing line diagram in peripheral CD4^+^ T cells of RA and healthy controls (HC).

Supplementary Figure 2 Mechanism diagram of Smad7 DNA methylation regulation in RA pathology. The binding of methyltransferases DNMTs to the promoter region of Smad7 inhibits the effect of demethylated MBDs, leading to the decreased expression of Smad7. The decreased expression of Smad7 affected the immune balance of CD4^+^ T cells though mediating aberrant Th17/Treg ratio, which leaded to the progression of RA.
