# Supplementary figures and images for: Hypermethylation of Smad7 in CD4+ T cells is associated with the disease activity of rheumatoid arthritis
Source: Front Immunol. 2023 Feb 9;14:1104881. doi: 10.3389/fimmu.2023.1104881 (PMC9947360; doi:10.3389/fimmu.2023.1104881)

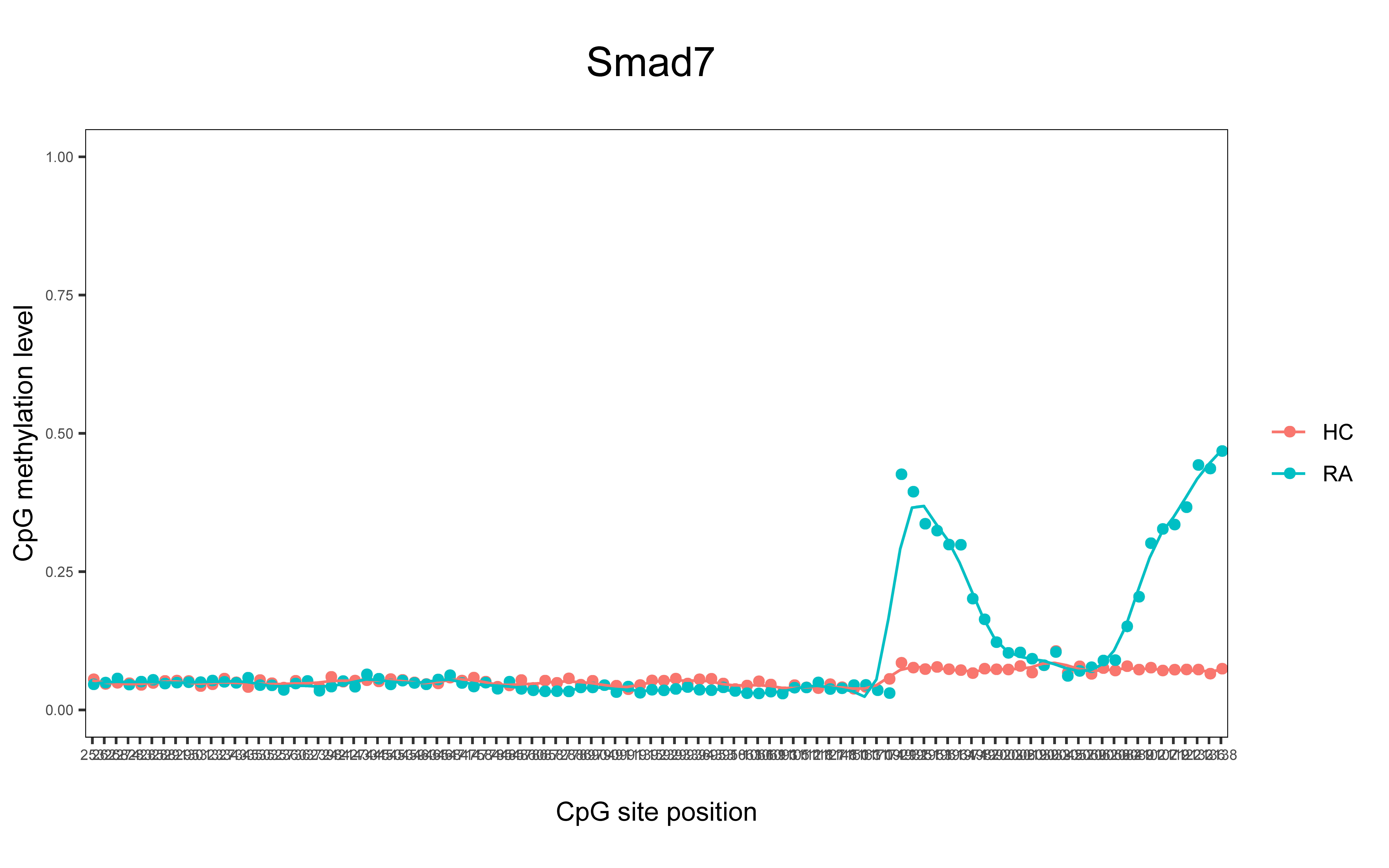

Supplement: Supplementary Figure 1 — Smad7 promoter methylation sequencing line diagram in peripheral CD4+ T cells of RA and healthy controls (HC). [file Image_1.jpeg]

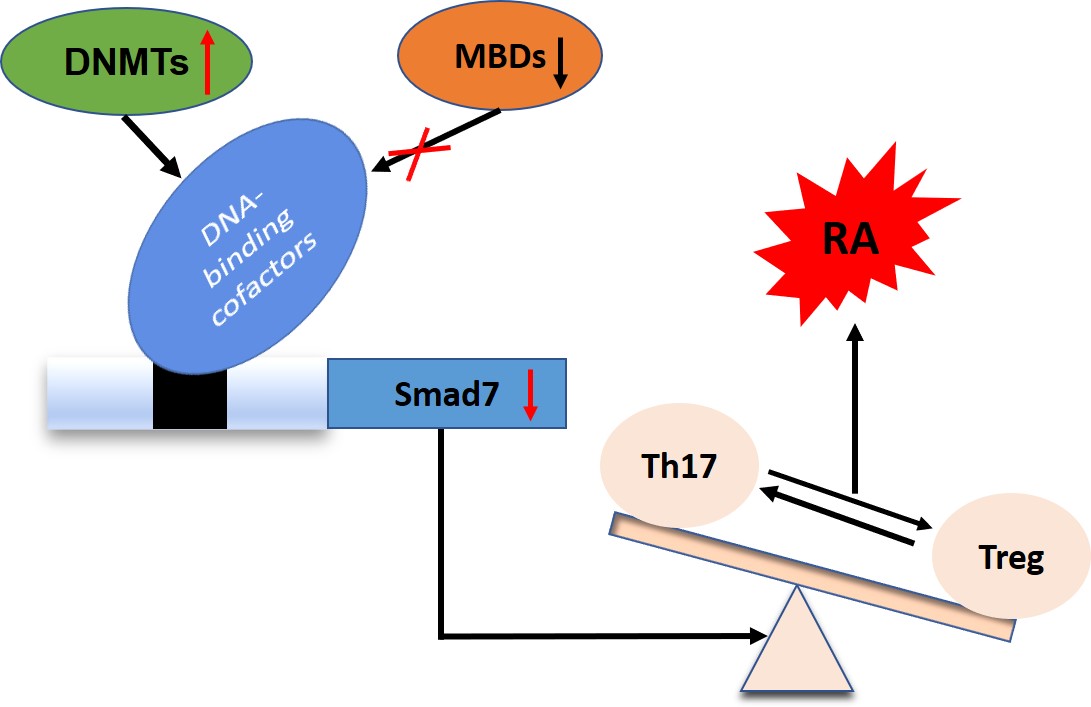

Supplement: Supplementary Figure 2 — Mechanism diagram of Smad7 DNA methylation regulation in RA pathology. The binding of methyltransferases DNMTs to the promoter region of Smad7 inhibits the effect of demethylated MBDs, leading to the decreased expression of Smad7. The decreased expression of Smad7 affected the immune balance of CD4+ T cells though mediating aberrant Th17/Treg ratio, which leaded to the progression of RA. [file Image_2.jpeg]
