# Supplementary material for: Hypermethylation of Smad7 in CD4+ T cells is associated with the disease activity of rheumatoid arthritis
Source: Front Immunol. 2023 Feb 9;14:1104881. doi: 10.3389/fimmu.2023.1104881 (PMC9947360; doi:10.3389/fimmu.2023.1104881)
Supplement: Supplementary file 3 [file Table_1.docx]

**STABLE 1. Demographic and clinical characteristics of RA patients.**

|  | RA (n=57) HC (35) |
| --- | --- |
| Age (years) | 50.04±13.76 47±12.43 |
| Gender (female) | 46 (80.70%) 32 (91.43%) |
| Disease course (years) | 5 (2, 10) / |
| ESR | 42.11± 21.66 / |
| CRP, mg/L | 9.44 (3.93, 30.29) / |
| IL-6, pg/mL | 24.57 (7.58, 47.85) / |
| Serum RF, g/L | 68.95 (23.98, 185.9) / |
| Serum anti-CCP, pg/mL | 132.9 (25.80, 200.00) / |
| WBC, 10^9^/L | 6.14(5.05, 6.95) / |
| Lymphocyte, 10^9^/L | 1.72(1.32, 2.06) / |
| CD4^+^ T % in lymphocyte | 47.39(41.65, 51.23) / |
| Platelet, 10^9^/L | 286.5 (250.5, 344.8) / |
| DAS28-CRP | 4.32 ± 1.10 / |
| Medication (%) |  |
| GCs | 38.6% |
| HCQ | 12.3% |
| MTX | 45.6% |
| LEF | 26.3% |
| SSZ | 3.5% |
| Cs | 1.8% |
| bDMARDs | 8.8% |
| NSAIDs | 63.2% |

**Note:** Statistic analysis data were expressed as median (centile 25, centile 75). Age, ESR level and DAS28-CRP were expressed as mean ± SD. RA, rheumatoid arthritis; ESR, the erythrocyte sedimentation rate; CRP, C-reactive protein; IL-6, interleukin-6; RF, rheumatoid factor; anti-CCP, antibodies to cyclic citrullinated peptide; WBC, white blood cell; DAS28-CRP, the 28-joint Disease Activity score calculated using CRP. GCs, glucocorticoids; HCQ, hydroxychloroquine; MTX, methotrexate; LEF, leflunomide; SSZ, sulfasalazine；Cs, Cyclosporin; bDMARDs, biologic disease modifying antirheumatic drugs; NSAIDs, Nonsteroidal anti-inflammatory drugs.
