# Supplementary material for: Hypermethylation of Smad7 in CD4+ T cells is associated with the disease activity of rheumatoid arthritis
Source: Front Immunol. 2023 Feb 9;14:1104881. doi: 10.3389/fimmu.2023.1104881 (PMC9947360; doi:10.3389/fimmu.2023.1104881)
Supplement: Supplementary file 4 [file Table_2.docx]

**STABLE 2. The primers sequences of BSP analysis**

| amplicon name primer sequence (5′-3′) |
| --- |
| Smad7-a left_primer GTTATTTGAATATTTTGTATAGTAGGAGGGGGA  right_primer AAATACCAAAAATCACCACCATCCCCAC  Smad7-b left-primer GTAGGYGATAGTAGTAGTAGTAGGGGTT  right_primer CTTTTTCCTTCTTCCAACAACCCAACC  Smad7-c left-primer GGGTATAAGTTGTTTGTTAGTGTAGGGGT  right_primer AACRCCCRACCCCTAAACCCCTAAATAC |
