# Supplementary material for: Hypermethylation of Smad7 in CD4+ T cells is associated with the disease activity of rheumatoid arthritis
Source: Front Immunol. 2023 Feb 9;14:1104881. doi: 10.3389/fimmu.2023.1104881 (PMC9947360; doi:10.3389/fimmu.2023.1104881)
Supplement: Supplementary file 5 [file Table_3.docx]

**STABLE 3. The primer** **sequences of qRT-PCR**

| Gene primer sequence (5′-3′) |
| --- |
| Smad7 Forward GAATCTTACGGGAAGATCAAC  Reverse CGCAGAGTCGGCTAAGGT  DNMT1 Forward TCCATGTCTGTTACTCGCCT  Reverse CAAGGCCACAAACACCATGT  DNM3A Forward CAATGACCTCTCCATCGTCAAC  Reverse CATGCAGGAGGCGGTAGAA  DNM3B Forward CTGCCGGTGTTTCTGTGTGG  Reverse TGTAACAGCTCCAGGGCTCC  MBD2 Forward ACGAATGAATGAACAGCCACG  Reverse TGCTACCTGGACCAACTCCT  MBD4 Forward CGAATGACCTCCGCAAAGAA  Reverse TCTTCCTGCTGTCTTCCCAA  GAPDH Forward CTGACTTCAACAGCGACACC  Reverse TAGCCAAATTCGTTGTCATAC |
